# Consequences of Ultra-Violet Irradiation on the Mechanical Properties of Spider Silk

**DOI:** 10.3390/jfb6030901

**Published:** 2015-09-10

**Authors:** Wee Loong Lai, Kheng Lim Goh

**Affiliations:** School of Engineering, Monash University Malaysia, Bandar Sunway 46150, Malaysia; E-Mail: weeloong88@gmail.com

**Keywords:** strength, extensibility, toughness, bond scission, bond crosslinks

## Abstract

The outstanding combination of high tensile strength and extensibility of spider silk is believed to contribute to the material’s toughness. Thus, there is great interest in engineering silk for biomedical products such as suture or implants. Additionally, over the years, many studies have also sought to enhance the mechanical properties of spider silk for wider applicability, e.g., by irradiating the material using ultra-violet radiation. However, the limitations surrounding the use of ultra-violet radiation for enhancing the mechanical properties of spider silk are not well-understood. Here, we have analyzed the mechanical properties of spider silk at short ultra-violet irradiation duration. Specimens of spider silk were subjected to ultra-violet irradiation (254-nm wavelength, i.e. UVC) for 10, 20, and 30 min, respectively, followed by tensile test to rupture to determine the strength (maximum stress), extensibility (rupture strain), and toughness (strain energy density to rupture). Controls, *i.e.*, specimens that did not received UVC, were also subjected to tensile test to rupture to determine the respective mechanical properties. One-way analysis of variance reveals that these properties decrease significantly (*p* < 0.05) with increasing irradiation duration. Among the three mechanical parameters, the strength of the spider silk degrades most rapidly; the extensibility of the spider silk degrades the slowest. Overall, these changes correspond to the observed surface modifications as well as the bond rupture between the peptide chains of the treated silk. Altogether, this simple but comprehensive study provides some key insights into the dependence of the mechanical properties on ultra-violet irradiation duration.

## 1. Introduction

The current challenge in material engineering is the production of light-weight materials with high strength, extensibility, and toughness. So far, naturally occurring materials such as spider silks are able to meet these demands: spider silk is strong [1,2] with a specific strength that is five times that of steel or two times that of Kevlar [3,4]. The combination of high strength and extensibility makes them the toughest material known to-date [5]. As biomaterials, spider silk has far-reaching biomedical applications [5,6]; it can be processed into films and scaffolds to improve tissue regeneration in skin, nerve, bone, and cartilage [6,7,8] or to repair ruptured connective tissues such as tendons and ligaments [5,9,10]. These films and scaffolds can provide structural support for the cells when the cells are seeded onto them [7]. Blending spider silk with less expensive synthetic polymers is one approach to reduce the cost of the biomaterials for, e.g., tissue regeneration applications [7]. Additionally, since the recombinant spider silk proteins have the ability to repair damaged nerves and tissues, it makes sense to explore novel wound dressings made from spider silk protein [10].

**Figure 1:**
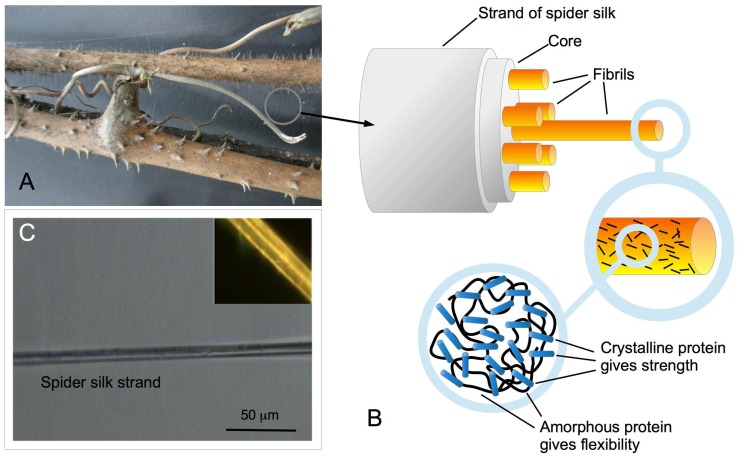
Spider silk. (**A**) A spider web attached to the branches of an outdoor plant; (**B**) Schematic of the hierarchical structure of spider silk; (**C**) A spider silk specimen mounted on a horizontal mechanical (tensile) tester available in the authors’ laboratory. Of note, the gap in the middle of a single strand silk suggests that the specimen comprises more than two strands.

The process of spinning silk fibre by a spider involves solidifying a non-Newtonian solution of protein as it flows through a narrow tube towards the spinneret. As partial crystallization of the protein molecules occurs, hydrogen bonds between the protein molecules facilitate the formation of beta sheets [11]. These beta sheets generate cross-links between protein molecules, resulting in the silk structure [11]. The crystalline region is hydrophobic and this region loses water during the silk solidification process; this also explains why the spider silk is insoluble to water [11]. Finally, when the solid state is achieved, we find that it possesses a hierarchical structure (Figure 1) [5]. Starting at the individual strand (which is observed at the microscopic level), it comprises a bundle of fibrils; at the next lower level, *i.e.*, fibrils, we find that each fibril contains proteins which are in crystalline or amorphous states [12,13].

However, spider silk is not easy to engineer: farming spider for large-scale production of silk is not feasible due to (1) the low production rate of silk by spiders [6] and (2) the cannibalism of spiders [14]. One of the ways to reproduce spider silk proteins and spin them under controlled *in vitro* conditions is forced silking, which involves collecting the silk fiber by a rotating cylinder while the spider is immobilized [15,16,17,18]. Since forced silking results in a very low yield, it is not useful as a large-scale manufacturing process.

In principle, it should be possible to enhance the mechanical properties of spider silk by inducing cross-links to form in the biopolymers. Perhaps this could involve exposing the spider silk to ultra-violet (UV) radiation similar to the exogeneous crosslinking method for connective tissue therapy [19]—the method of UV crosslinking is also well-known in biomedical engineering for enhancing the mechanical properties of biomaterials [20]. The key issues underlying the interaction of UV radiation with tissue address: (1) the dependence of the absorbed UV photon energy, ξ, per unit mass of the tissue on the exposure time and UV wavelength; (2) an intervening thermodynamic shear-related parameter, *G*, that quantifies the extent of UV-induced cross-linking in the tissue; (3) a threshold model for the *G versus* ξ relationship, characterized by *t_C_* and (4) the role of *G* in influencing the tissue elasticity [19]. On the basis of these arguments, a framework has been proposed to predict the implicit dependence of UV-induced crosslinks on the exposure time (up to 60 min) and UV wavelength, namely 254 nm (which falls within the UVC range, *i.e.*, 200–280 nm) and 365 nm (which falls within the UVA range, *i.e.*, 315–400 nm), for understanding how UV directs the tissue stiffness and resilience [19]. As for spider silk, it has been shown that the formation of new crosslinks between chains [21] occurs when spider silk is exposed (namely UVA, at wavelength 365 nm) within five hours, and consequentially an increase in tensile strength is observed [18,22]. However, cleavage of protein chains by UVA occurs when spider silk is exposed to long irradiation duration (>30 h), and consequentially this leads to a decrease in tensile strength [18,22,23,24] and extensibility [25].

Although UVC (wavelength: 200–280 nm) is also known to cause the diminution in the mechanical properties of spider silk [22], so far these studies have shown that it occurs within hours [22]. Unfortunately, the effects at very short irradiation durations (*i.e*., minutes) are not well understood. We hypothesize that short irradiation durations using UVC will lead to enhanced mechanical properties for the spider silk. Here, we report our findings from a simple study of the influence of UVC on the tensile properties of spider silk at short irradiation duration (on the order of minutes). Why, then, are we concerned with the UV irradiation of spider silk when it would have been exposed to UV radiation in the environment [25]? The reason is that the UV radiation that reaches the Earth is mainly UVA [22] and (5% of the total UV radiation) UVB (280–315 nm) while a large proportion of the radiation, e.g., UVC, would be attenuated by the ozone layer before reaching earth [22]—this implies that naturally occuring spider silk would not have been exposed to UVC in the environment.

## 2. Results

### 2.1. Microscopy

Figure 1C shows a typical image of the silk fiber under a light microscope. A simple examination reveals that there is no appreciable differences in the reflectivity property between the controls and the UV treated specimens at the respective irradiation duration.

**Figure 2:**
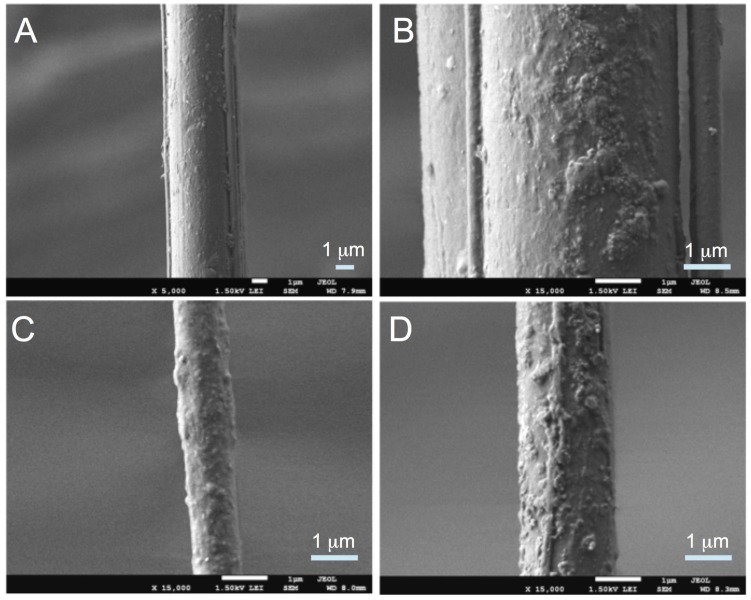

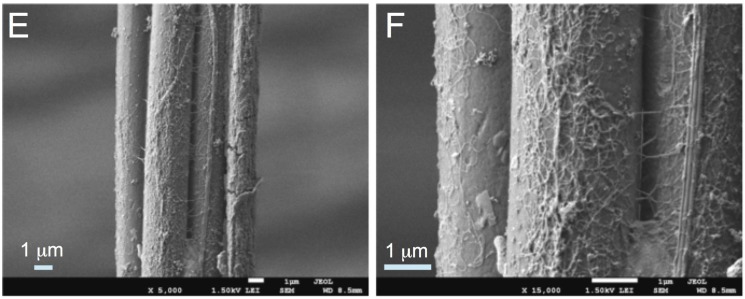
SEM images of spider silk. The controls are shown in (**A**,**B**) at two different magnifications, namely ×5000 and ×15000, respectively. The specimens that are irradiated at 10 min and 20 min are shown in (**C**,**D**), respectively. The images in (**E**,**F**) are from specimens irradiated at 30 min, shown for two different magnifications, namely ×5000 and ×1500, respectively.

Examination of the SEM images of the untreated specimens at low magnification reveals a relatively smooth texture over a large proportion of the surface of each strand (Figure 2A). On closer examination at higher magnification, randomly dispersed small, near-spherical bumps and large irregular bumps with interconnections between the bumps were observed; overall no depression-like features were observed (Figure 2B). However, a greater proportion of the surface of the UV treated specimens appears to be covered by aggregates of small compact masses (Figure 2C–E) compared to the untreated specimens. These small compact masses appear near-spherical in shape with interconnections between the masses (as reveal in the specimens treated to 10 and 20 min UV irradiation, see Figure 2C,D). Curiously these masses appeared to disappear appreciably at 30 min UV irradiation, only to be replaced by a fine mesh-like network with no obvious preferred orientation nor aggregation.

### 2.2. Micro-Tensile Test

**Figure 3:**
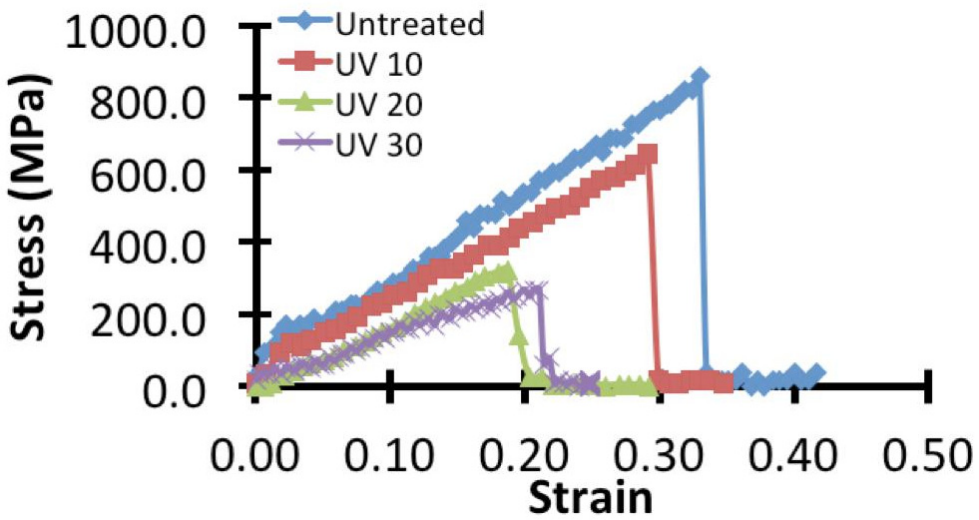
Typical stress strain curves of spider silk specimens from the control group and the UV treated groups (10, 20 and 30 min).

Figure 3 shows typical stress-strain curves from specimens derived from the controls and the respective UV treated groups. The general profile of the stress-strain curve features a steep slope at initial loading; there exist a point on the curve beyond which the slope is appreciably reduced. The highest stress point corresponds to specimen rupture. During initial loading, the stress-strain slope corresponding to the control specimen is steeper than that treated for 10 min UV irradiation. However, for the specimens irradiated at 20 and 30 min respectively, the difference between the slope of the region corresponding to initial loading and the slope at later loading is less appreciable. Overall, the maximum stress and rupture strain are highest for the control specimen and appear to decrease with increasing UV irradiation duration; this also implies that the fracture toughness (*i.e*., the strain energy density to rupture) decreases with increasing UV irradiation duration.

**Figure 4:**
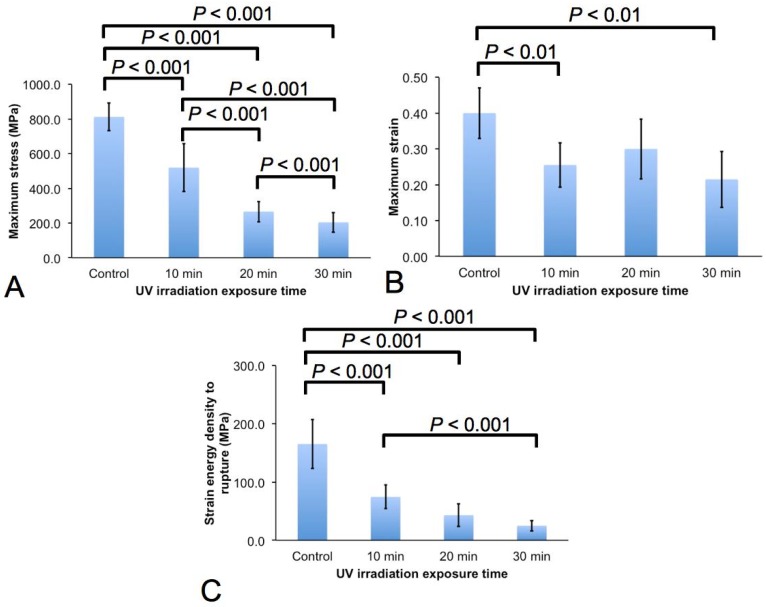
Mechanical properties of the spider silk. Graphs of (**A**) maximum stress and (**B**) maximum strain and (**C**) strain energy density to rupture, *versus* the respective UV treatment levels (as well as the control group).

One-way analysis of variance (ANOVA) reveals significant differences (*p* < 0.05) in the maximum stress, rupture strain, and the strain energy density to rupture (Figure 4). This indicates that there is strong evidence to conclude that not all the means (*i.e*., associated with the different UV irradiation durations and with the control group) of the respective mechanical parameter are equal when the level of significance is set at 0.05. In particular, *post-hoc* Tukey test yields four sets of confidence intervals (CIs) for the respective mechanical parameters. Pair-wise multiple comparisons of the CIs for those that exclude zero lead to the following results. For the maximum stress, significant differences are observed in the magnitudes of the maximum stress between the controls and the respective UV treated groups; significant differences are also observed between the respective UV treated groups. For the rupture strain, while significant differences are observed in the magnitudes of the rupture strain between the controls and the 10 min and 30 min groups, no significant difference is observed in the magnitudes of the maximum strain between the controls and the 20 min groups. Accordingly, no significant difference is also observed in the magnitudes of the maximum strain between the UV treated groups. For the strain energy density to rupture, while significant differences are observed in the magnitudes of the strain energy density to rupture between the controls and the respective UV treated groups, only the 10 and 30 min groups yield significant differences in the magnitude of the strain energy density. Accordingly no significant difference is observed in the magnitude of the strain energy density between 10 and 20 min groups as well as between 20 and 30 min groups.

### 2.3. Biochemical Compositional Analyses

**Figure 5:**
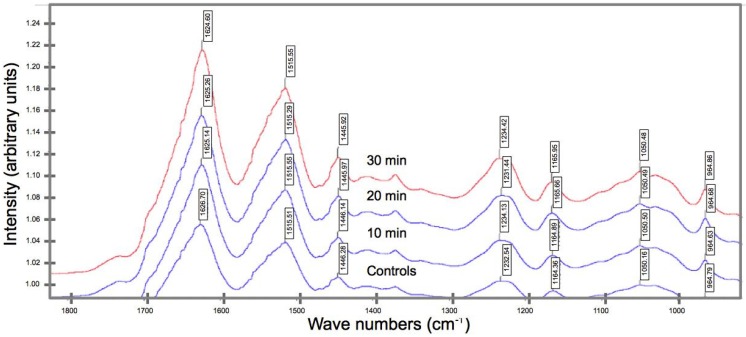
FT-IR spectra of spider silk specimens from the control group and the respective UV irradiated groups. Each spectrum was obtained by averaging the spectra of the specimens within each group.

We compare the (average) spectrum of specimens from the control group and UV irradiated groups (Figure 5). We find that (1) the peaks falling within the range of wavenumbers, namely 1700–1600 cm^−1^, are attributed to the Amide I; (2) the peaks falling within 1600–1480 cm^−1^ are attributed to Amide II; (3) the peaks falling within 1350–1190 cm^−1^ are attributed to Amide III [26]. Other amino acid side chains could also contribute to the peaks within these ranges, *i.e.*, 1480–1350 and 1190–700 cm^−1^ [26]. There were no new peaks and no new functional groups after the spider silks were UV treated. More importantly, the wavenumber of the peak Amide I appears to shift to a lower value with increasing UV irradiation duration; in particular, the wavenumber of the peaks corresponding to the controls (1626.7 cm^−1^) wavenumber decreases to 1624.6 cm^−1^ after a 30-min irradiation regime.

## 3. Discussion

### 3.1. Effects on the Mechanical Properties

The changes to the surface on the spider silk may be attributed to the broken polypeptide chains [27]—polypeptide chains break up when the hydrogen bonds between the polypeptide chains are removed by high-energy UV radiation (such as UVC). Prolonged irradiation (~30 min) increases the proportion of polypeptide chains break up within aggregates of polypeptides in the spider silk. Thus the small compact masses covering the spider silk surface as shown in the SEM images (Figure 2) could be attributed to aggregates of broken polypeptides on surface of the spider silks. Nevertheless, given the attenuating nature of UVC, it is expected that the effects are not just confined to the surface but also within the spider silk.

UV irradiation of spider silk leads to a reduction in strength, extensibility and fracture toughness. Between the untreated and UV treated (to 30 min irradiation) spider silk, we find that (1) the strength of the former is four times that of the latter; (2) the extensibility of the former is about twice that of the latter; (3) the fracture toughness of the former is five times that of the latter. To answer the question of which mechanical parameter degrades fastest under UV exposure, we carried out an order of magnitude estimates of the rate of degradation of the respective mechanical properties by modelling the respective mechanical parameter as a function of irradiation duration using an exponential function, *f* = *K_f_*exp(−*t*/τ*_f_*), where *f* represents the maximum stress, rupture strain or strain energy density to rupture, *t* the exposure time (*t* = 0 corresponds to the untreated specimens), *K_f_* the mechanical property of the untreated specimens and finally, τ*_f_* the time constant. We note that τ is an important parameter as it is related to the response of the respective mechanical property of spider silk to UV irradiation; thus *f* = *K_f_*/*e* when *t* = τ*_f_*. Accordingly, by a simple curve-fitting procedure, we find the estimates for τ*_f_* as follows, in order of decreasing value: 52 min (rupture strain), 24 min (strain energy density to rupture), and 20 min (maximum stress). Thus, the strength of the spider silk degrades most rapidly; the extensibility of the spider silk degrades slowest. Interestingly, the degradation rate for fracture toughness lies somewhat in between those for strength and extensibility.

The strength, extensibility and fracture toughness of spider silk from different spider species are expected to vary, depending on the biochemical composition of the different amino acids [28]. In this study, the untreated *Araneus diadematus* spider silk specimens yield a mean strength, extensibility and fracture toughness of 0.8 GPa, 0.40, and 150 GPa, respectively (Figure 4). Spider silks from *Araneus seriaticus* yield tensile strength, extensibility, and fracture toughness of 1.1 GPa, 0.24, and 158 GPa, respectively (when tested at a strain rate ~0.024 s^−1^); *Nephila clavata* spider silks yield higher tensile strength (about 2 GPa) and extensibility (about 0.30) (strain rate ~0.02 s^−1^) [25]. It is also likely that the spider silk shows different properties depending on where the fiber is secreted; the strength of viscid silk produced in the flagelliform glands is 0.5 GPa, which is lower than the fibers from the major ampullate glands [29]. Finally, although the UVC irradiation approach has failed to enhance the mechanical properties of the spider silk, the range of magnitudes for the fracture strength, extensibility, and fracture toughness overlaps with the corresponding mechanical properties of some of the respective hierarchical levels of the ligaments and tendons [30,31,32]. From a mechanical compatibility perspective, this suggests that the UVC irradiation method for spider silk may be exploited for developing spider silk bioscaffold implants for repairing ligaments and tendons.

It is important to note how the synthesis process contributes to the mechanical properties of the spider silk. During the spinning process, the liquid protein solution flows through the spinning duct, in the presence of external stresses (exerted by the spider) applied to the solution [3]. Consequently, defects such as dislocations will start to develop in the solid crystal; by an analogy to how metal deforms by dislocation movement, these defects promote high tensile strength of the spun thread [3]. Additionally, the properties of the final spun silk also depended on how the liquid flows through a narrow tube towards the spinneret: the protein molecules undergo partial crystallization and hydrogen bonds are formed between the beta sheets, thus strengthening the protein molecules further [3]. Furthermore, the high stress generated during this processing stage causes the realignment of these protein molecules, resulting in a more extended conformation so that the protein molecules could be bridged via hydrogen bonds (analogous to zip fasteners) to give the anti-parallel beta conformation of the final thread.

### 3.2. Effects of UV on the Biochemical Composition

UV light is a well-known promoter of free radicals in protein molecules [19]. These free radicals will react with the other amino acids in the protein molecules to form new crosslinks and this will result in an increase in the crosslink densities in the protein molecules [19] but it could also lead to bond scission [19,22]. As pointed out in Section 2.3, with reference to the FT-IR spectrum, the Amide I band reveals a decrease in wavenumber from 1626.7 cm^−1^ (controls) to 1624.6 cm^−1^ (30 min UV). This may be attributed to bond scission (namely hydrogen bonds) by free radicals generated within the material when the molecules in the spider silk interact with the UV radiation. Of note, these hydrogen bonds are essential for maintaining the molecular structure.

The underlying basis for the FT-IR peaks that are identified to be associated with the amides are attributed to a vibrational mode contributed by proteins and polypeptides [33]. In particular, the peaks in the FT-IR spectra within the (1) 1700–1600 cm^−1^ is likely to be attributed to the Amide I, due to stretching of the C=O; (2) 1600–1480 cm^−1^ is likely to be attributed to Amide II (due to the coupling of N-H in-plane), also due to the stretching of C=O; (3) 1350–1190 cm^−1^ is likely to be attributed to Amide III, due to the C=N stretching, coupled to the in-plane N-H bending mode [33]. The peaks in the other bands, namely 1480–1350 and 1190–700 cm^−1^ are attributed to the existence of other amino acid side chains [33]. Further analysis of the band 1600–1480 cm^−1^ reveals that (a) α-helical structures are responsible for the range 1660–1652 cm^−1^ [34]; (b) polyglycine II-like structures (having an amide I absorption and a typical amide II band at 1550 cm^−1^) could be responsible for the range 1658–1663 cm^−1^ [35]; (c) the β-sheets could be responsible for the range 1670–1695 cm^−1^ (as well 1610–1640 cm^−1^) [26].

### 3.3. Implications on the Structure-Function Relationships

The structure-property relationship describes the spider silk as a strong, extensible and tough nanostructured material which may be attributed to the presence of ordered (*i.e*., crystalline) and disordered (*i.e*., amorphous) phases within the proteins in the fibrils (Figure 1) [13]. Overall, spider silk is predominated by polyalanine, which forms strong beta-sheet nanocrystals that are assembled by weak inter-strand H-bonds [13]. These crystals are embedded in an amorphous, glycine-rich matrix that consists primarily of random coils and helices and hence has lower rigidity [13]. This combination of crystalline and amorphous phases leads to a spider silk with exceptional mechanical properties, namely high extensibility, strength and fracture toughness. The two-phase structure plays a role in ensuring a cohesive network that can store and dissipate energy in the spider silk [13]. The mechanical failure of the spider silk is linked to the crystals in the crystalline phase. The size of the crystals may have an important role in influencing the spider silk mechanical properties, by an analogy to the strengthening of polycrystalline material based on the well-known Hall-Petch model in materials science, which describes how the ideal yield strength of the material is modified by a structure-related term that is inversely proportional to the square-root of the crystallite size. Naturally occurring spider silk possesses crystal size ~2.5 nm [36]. It is suggested that further enhancement to the silk mechanical property (*i.e*., yield strength) can been achieved by decreasing the crystallite size to 2.1 nm [36]. Conversely, the larger the crystallite the smaller is the yield strength of the spider silk. Nevertheless, as it is not clear how UV irradiation may result in large crystallites, this could be a subject for further study.

In principle, the effect of UV radiation on spider silk is to excite the electrons in the material. Electron-excited molecules (and atoms) will alter the kinetics of chemical reactions, resulting in chemical reactions that promote crosslinking (or chain scission) in the material structure [24]. As pointed out in previous paragraph, in the structure-function relationship of the spider silk, the polyalanine crystalline phase contains β-poly (l-alanine) [37]; the amorphous phase contains crosslinked beta-pleated sheet, amorphous network chains, polyalanine blocks and residue glycine-rich blocks [37]. Indeed, the overall amorphous region may be regarded as semiextended, well-oriented, and more sparsely H-bonded structures that resemble three-fold helices [13]. The polyalanine segments in the crystalline regions are responsible for the silk’s tensile strength while the glycine residues in the amorphous regions are responsible for spider silk’s extensibility [13]. The increase in crosslinks in the spider silk as a result of UV irradiation may occur in the beta-pleated sheet in the amorphous phase and the β-poly (l-alanine) in the crystalline phase. To order of magnitude, estimates of the fracture strength of the spider silk may be derived from a simple model given by, σ = σʹ − *C*/*M*, where σ is the modified fracture strength of the spider silk, σʹ the ideal fracture strength, *M* the molecular weight of the polymeric molecules and *C* is constant. Consequently, the crosslinks generated by the UV irradiation would result in a higher net molecular weight of the beta-pleated sheet in the amorphous phase and the β-poly (l-alanine). This in turn leads to a value for the term, *C*/*M*, that is smaller than that associated with the control. These arguments lend themselves to predicting the augmentation of the σ. Conversely, scission of these crosslinks then result in a lower *M* and consequently leads to the dimunition of the σ.

**Figure 6:**
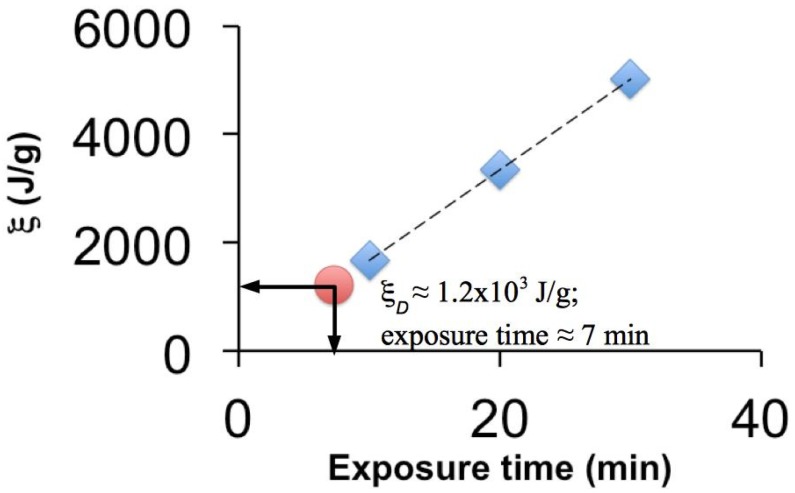
Predicted energy absorbed, ξ, *versus* exposure time during UVC irradiation derived from a thermomechanical model [19]. The diamond symbols correspond to 10, 20, and 30 min exposure times. The circular symbol corresponds to the threshold point, *i.e.*, when ξ = ξ*_D_* ≈ 1.2 × 10^3^ J/g at an exposure duration of seven minutes for chain scission.

The diminution of the mechanical properties of the spider silk subjected to prolonged UV exposure suggests a possible energy threshold, ξ*_D_* (similar to previous studies on irradiation of connective tissues by UV [19]), above which chain scission reaction predominates within the beta-pleated sheet in the amorphous phase and the β-poly (l-alanine) in the crystalline phase. Let η represents the electron density of the micro-environment in the spider silk, α the molecular absorption cross-sectional area, *I_r_* the UV intensity at distance, *r*, from the source, ρ/μ the specific surface area, defined as the ratio of exposed surface area (ρ) to the mass of the specimen (μ), Ω the radiomechanical photosensitizer efficiency and ξ the UV photon energy per unit of mass absorbed by the spider silk. On the basis of the justification used to predict the values of the η, α, *I_r_*, ρ/μ, and ξ for connective tissues [19], and of Ω (Section 4.2), we present an order of magnitude estimate for ξ associated with the respective exposure times. In general, we note that the intensity of the source, *I*_0_, is estimated as equal to the preset UV intensity and *r* as equal to the height of the chamber (Section 4.1). Given that the UV wavelength λ = 254 nm, step 1, we substituted λ into an electron density equation [19] to determine η (we find η = 1.4 × 10^24^ cm^−3^). Step 2, we determined α using an exponential equation that describe α as a function of the λ [19] (we find α = 1 × 10^−28^ cm^2^). Step 3, from the estimates of ρ (≈5.6 × 10^−12^ cm^2^) and μ (=0.02 mg), we determined ρ/μ (we find ρ/μ = 2.8 × 10^−7^ cm^2^/g). Step 4, from the estimates of *r* and *I*_0_, we substituted (together with the predetermined values of η and α) into the Beer-Lambert equation [19] to determine *I_r_* (we find *I_r_* = 0.0598 J·cm^−2^·min^−1^). Step 5, we provided an order-of-magnitude estimate (that would be consistent with the energy absorbed that is associated with the shift in the Amide peaks) for Ω for UV-irradiated photosensitizer-impregnated spider silk; this works out to be of the order of 10, *i.e*., Ω = 10^10^. Step 6, we evaluated ξ by noting that it is equal to the product of *I_r_* with ρ/μ and the respective exposure times [19]—then we multiplied the ξ by Ω for photosensitizer-impregnated spider silk to obtain the net energy absorbed per unit mass of the specimen, for the respective exposure times. Figure 6 shows a plot of ξ *versus* exposure times. Thus the model predicts that the threshold for chain scission actually occurs at about seven minutes.

## 4. Experimental Methods

### 4.1. Ultra-Violet Irradiation of Spider Silks

Spider silks were harvested from an entire web (made by an *Araneus diadematus* spider) that was attached to the branches of an outdoor garden plant. For treatment by UV irradiation, specimens were placed in a UV machine (UV Crosslinkers CL-1000, UVP); the specimen chamber (12.7 (*H*) × 30.5 (*W*) × 25.4 (*D*) cm) contained five discharge-type tubes (8 W/tube, wavelength 254 nm) located on the chamber roof. The specimens were placed in a glass petri-dish on the chamber floor at approximately 30.5 cm away from the middle discharge tube. Glass petri dish was used for holding the specimens during irradiation instead of plastic petri dish to prevent any chemical reaction with the specimen arising from the UV radiation. The dish was not covered to avoid attenuating the UV radiation. A preset UV intensity of 0.06 J/cm^2^ min was used.

The web was separated into four groups (Table 1). Three groups were subjected to UV irradiation corresponding to three levels of irradiation time (10, 20, and 30 min). The fourth group, *i.e.*, a control group (untreated specimens), was established for the purpose of comparison. The spider silk in each group was dissected further into small sections; thus each treatment group comprised ten (sections) specimens. Each section had a length of 8 cm; the mean thickness of the specimens from control and the treatment groups ranged 7.0–11.5 μm. Of note, each section comprised several strands of silk fibres. Ten sections were randomly selected from each group to be used as test specimens. Thus, a total of 40 test specimens were prepared according to the three levels of irradiation time and the control group.

**Table 1:** Specimen thickness for the respective treatment groups.

Specimen Number	Control (μm)	UV Treatment		
		10 min (μm)	20 min (μm)	30 min (μm)
1	17.8	6.9	6.1	6.5
2	6.5	4.0	7.3	10.5
3	4.6	7.5	11.2	7.3
4	10.4	9.8	11.2	8.5
5	6.8	7.0	7.2	10.2
6	3.3	10.3	7.6	6.2
7	4.5	9.2	31.3	9.6
8	3.6	9.3	16.2	12.6
9	5.8	9.8	11.9	12.8
10	7.2	11.4	5.5	8.0

### 4.2. Physical Examination and Chemical Characterization

The morphology of all the (controls and UV irradiated) specimens was examined using both light microscopy (Nikon Eclipse TS100) and a high-resolution JEOL JSM-7600F field-emission SEM. For the SEM imaging, the specimens were not coated to avoid any artifacts arising from coating effects. Since no coating was applied to the specimens, the imaging could only be done at a low kV (1.5 kV) to avoid damaging the specimens. Fourier transform infrared spectroscopy (FT-IR) (FTS3100 Varian Inc., Palo Alto, CA, USA) of the compositional characteristics of the functional groups of the spider silk (controls and UV irradiated) was carried out using an approach described elsewhere [30]. Here, approximately 2 mg of each sample was embedded in 100 mg KBr and the mixture was pressed into discs. The FT-IR sample compartment was continuously purged with dry air to prevent the formation of water vapor. All spectra were obtained from 400 to 4000 cm^−1^ with 36 scans per specimen, at 4 cm^−1^ resolution, and averaged to obtain a representative plot. Background subtraction was carried out by software.

### 4.3. Micro-Tensile Tests

**Figure 7:**
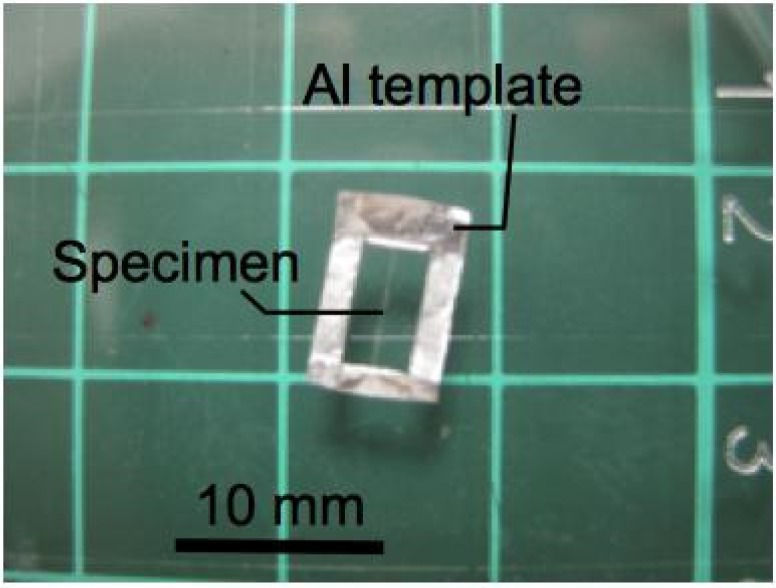
A spider silk specimen secured onto an aluminum template.

A high-throughput small-scale horizontal tensile test rig (developed in-house) was used to determine the tensile properties of the spider silk. For a detailed description of the rig (including the versatility of the rig for related applications) see previous reports [19,30,38,39,40]. Each specimen from the four groups was mounted onto an aluminum template (Figure 7) using a cyanoacrylate adhesive; grip plates were used to secure the specimen-template on the rig.

Force (*F*) and grip-to-grip displacement (Δ) were recorded for each specimen; they were used to derive the corresponding nominal stress (=*F*/*a*) and strain (=Δ/*l*_o_). Here, *a* is the cross-section area of each specimen; *l*_o_ is the gauge length of the spider silk specimen which corresponds to the initial grip-to-grip distance, typically about 5 mm (adjusted until just before the specimen became taut). The specimen was stretched (Figure 1C) to rupture at a displacement rate of 0.06 mm/s (this is equivalent to a strain rate of (0.06 mm/s)/*l*_o_ = 0.012 s^−1^). From the stress-strain curve (Figure 3), the maximum stress, rupture strain, and strain energy density to rupture were evaluated to parameterize the spider silk strength, extensibility, and fracture toughness, respectively. In particular, the strain energy density to rupture, which is a parameter derived from the stress and strain data points, was determined from the strain at the origin to the rupture strain.

### 4.4. Statistical Analysis

As mentioned in Section 1, our hypothesis is that short UVC irradiation durations enhance the mechanical properties of spider silk. We will evaluate this hypothesis by testing the null hypothesis (UVC has no effect on the mechanical properties of spider silk) *versus* the alternative hypothesis. Here, we note that preliminary assessment of the stress-strain data obtained from the mechanical tests has revealed that the residuals follow a somewhat normal distribution with a somewhat uniform variance. Consequently, one-way ANOVA, complemented by Tukey’s comparison of the mean values (family error rate = 0.01), was used to test the hypothesis for the respective mechanical parameters to UV irradiation treatment for the following levels of UV exposure times: 0 (control), 10, 20, and 30 min. Statistical analysis was carried out using MINITAB (version 16). Differences due to the treatment were considered significant if the *p*-value >0.05.

## 5. Conclusions

This study has investigated the mechanical properties, morphological changes, and biochemical composition of spider silk exposed to UVC radiation for short irradiation durations (up to 30 min). The conclusions are as follows:

1. The spider silk experiences decrease in fracture strength, extensibility, and fracture toughness with increased irradiation duration. In particular with regards to the rate of degradation, it is found that the strength of the spider silk degrades most rapidly; the extensibility of the spider silk degrades the slowest.
2. UVC irradiation affects the surface morphology of the spider silks; small compact masses (attributing to fragments of the peptide chains) are observed to cover the surface, with interconnecting bridges.
3. FT-IR spectroscopy reveals a shift in the peak position of a key functional group from a higher to a lower wavenumber (for UV irradiated spider silk), suggesting the occurrence of bond rupture within the peptide chains, which in turn implicates the diminution in the strength, extensibility, and fracture toughness.

Although the irradiation of spider silk using UVC leads to diminution of the mechanical properties, we do not rule out the wider applicability of this technique for modifying the mechanical properties of the material. Interestingly, it may be possible to develop (UVC treated) scaffold implants made from spider silk for tissues that are characterized by lower strength, extensibility, and toughness.
